# Sanitary conditions, waste management, safety measures and sources of air pollution associated with shopping malls in Nigeria’s largest city

**DOI:** 10.1016/j.puhip.2023.100376

**Published:** 2023-02-24

**Authors:** Doris N. Omeokachie, Godson R.E.E. Ana, Temitope A. Laniyan, David B. Olawade, Olawale J. Abaire, Deborah T. Esan

**Affiliations:** a Department of Environmental Health Sciences, Faculty of Public Health, University of Ibadan, Nigeria; b Department of Biochemistry, Adekunle Ajasin University, Akungba-Akoko, Nigeria; c Department of Nursing Science, College of Medicine and Health Sciences, Afe Babalola University, Ado-Ekiti, Nigeria

**Keywords:** Shopping malls, Sanitary conditions, Waste management, Safety measures

## Abstract

**Objectives:**

Shopping malls are fast becoming one of the most visited public spaces globally. However, information on the possible environmental conditions in relation to health hazards in shopping malls is poorly documented in developing countries. This study assessed the sanitary conditions, waste management, safety measures and sources of air pollution associated with selected shopping malls in Nigeria.

Study Design: a descriptive cross-sectional study design was adopted using a comparative approach.

**Methods:**

Three shopping malls (Mall Q, Mall R, and Mall S) in urban areas in Ibadan, Oyo State, Nigeria, were selected using convenience sampling technique. Three major shopping malls were selected using convenience sampling technique. Fifty seven, thirty five, and twenty nine stores were sampled in Mall Q, Mall R, and Mall S respectively. Direct on-site built environment and sanitary conditions of shopping malls were assessed using an observational checklist.

**Results:**

It was observed that all the selected shopping malls had air vents that were free from dust, unbroken walls, and emergency exits, although mold growths were observed on the walls and ceilings of Mall Q and Mall R. Toilet facilities were present and functional across all the shopping malls. Waste management facilities were available across the shopping malls with the absence of overfilled waste bins as regular emptying of the waste bins was a routine. Also, various safety measures and equipment were utilized across all the shopping malls, but safety signals and smoke detectors were absent in Mall R. Furthermore, Mall R and S were 5 m within the proximity of major roads, parking lots and public drainage channels.

**Conclusions:**

These findings reveal a need for improvement in the hygiene and sanitary conditions within shopping malls. Hence, there should be periodic environmental monitoring, and proper housekeeping practices should be encouraged in shopping malls in Nigeria.

## 1 Introduction

Most people go to the mall for fun, relaxation, shopping and eating without giving a second thought to the environmental hazards of shopping malls. However, due to their enclosed space with huge crowd capacity, malls are faced with lots of health and environmental issues [1]. Before the Coronavirus disease (COVID-19) in December 2019, hygienic conditions in shopping malls were not taken as seriously as they should have been [2]. The World Health Organization (WHO) has put together certain health protocols as preventive measures for public place users, including the shopping mall. This includes the following:

• Performing hand hygiene frequently using alcohol-based hand sanitizer;
• Regular washing of your hands with soap under running water
• Avoiding touching one's eyes, nose, and mouth
• Wearing a mouth-and-nose mask
• Maintaining social and physical distance [3].

All of these became sanitary measures against the spread of diseases within public places and gatherings. Interestingly, many nations, including Nigeria, had to adopt the same sanitary strategy in all their public places. Likewise, the problem of solid, liquid, and toxic waste management in Africa, especially in Nigeria, has come with urbanization. An important feature of urbanization is the building of malls and massive edifices in metropolitan areas. Of course, the high rate of having many of these variety stores implies a rapid accumulation of refuse in and around the buildings. Improper waste disposal by one individual affects the entire citizenry. So, as a policy, many countries have tasked every individual, establishment, or institution to contribute significantly to the process of keeping their communities and environment clean [4,5]. But this is not so for many malls in Nigeria, as most of their waste overflows and spills all over the place, causing contamination to visitors to the malls. This research paper examines the sanitary conditions, waste management, safety measures and sources of air pollution associated with some selected shopping malls in Nigeria.

Consequently, indoor air quality has become a major health issue worldwide as the rising levels of outdoor air pollution keep increasing, making the air inside homes and enclosed facilities more toxic. This is why it is critical to know how mall air quality affects a person's health and well-being. Poor indoor air quality in a shopping mall will unquestionably cause discomfort to shoppers and affect their total wellbeing. In a situation where the indoor CO_2_ levels of a mall exceed 1000 parts per million, many occupants of the mall were reported to have developed symptoms of sickness, such as fatigue, irritation of the eyes, nose, and throat, and shortness of breath [6]. However, the air quality within malls has been the only concern lately. The sanitary condition, waste management, and the safe measures to correct all these menace within many malls need to be given attention to as well.

An example of environmental issues associated with malls is indoor air pollution. Since malls are normally located close to main roads in urban cities, vehicle exhausts, dusts, concentrations of Carbon dioxides, and several other pollutants affect the quality of air in malls [7]. Many human activities, such as burning wood for fuel, mining, powering commercial and residential buildings with fossil fuels, and driving automobiles and mining, contribute to urban air pollution in Sub-Saharan African countries like Nigeria [8]. Because many shopping centers are built next to busy roads, their indoor air quality is contaminated and, as a result, dangerous to people's health. Subsequently, increased urbanization is associated with urban air pollution problems. As the population of cities grows, so does the pollution in the environment, soil, water and air, which contribute to acidification [9]. The poor air quality inside many shopping malls is mostly due to the different products and chemical substances within the mall as well as the environmental pollution around the hall premises [10].

The objective of this research is to investigate the current sanitary conditions, waste management practices, safety measures, and sources of air pollution associated with shopping malls in Nigeria's largest city. The study aims to identify the extent of compliance with relevant regulations and guidelines, assess the environmental and health impacts of these conditions, and propose evidence-based recommendations to enhance the sustainability and livability of shopping malls in the city.

## 2 Methodology

### 2.1 Study design

The study is a descriptive cross-sectional design with a comparative approach which entailed direct on-site observations of sanitary conditions, waste management, safety measures and sources of air pollution of selected shopping malls. Three major shopping malls were selected using convenience sampling technique. Fifty seven, thirty five, and twenty nine stores were sampled in Mall Q, Mall R, and Mall S respectively.

### 2.2 Study locations

The study locations were located in the urban region of Ibadan which comprised of five local government areas- Ibadan North, Ibadan South East, Ibadan South West, Ibadan North East and Ibadan North West, in which three of the aforementioned local government areas were selected. However, the study locations were selected based on the following defined criteria:

### 2.3 Inclusion criteria for the study location

1. The local government area to be selected must possess a shopping mall.
2. Shopping malls to be used are defined as enclosed buildings with shops representing merchandisers with interconnecting walkways that enable customers to walk from unit to unit with a central management.

### 2.4 Exclusion criteria for the study location

1. Local government areas without shopping malls were not selected

### 2.5 Description of the selected local government areas

Ibadan North Local Government Area has been known to be one of the largest local government areas in Urban Ibadan which consist of 12 wards. It was created in 1991 with a population of 856,988 inhabitants and covers a landmass of 420 km^2^ with its headquarters situated in Agodi-Gate. However, Ibadan South West Local Government Area was created in 1991 which is made up of 12 wards and boasts of a population of 283,098 inhabitants and its headquarters is situated in Ring-Road, Ibadan. While Ibadan North West Local Government Area which is comprised of 11 wards and has a population of 152,834, covers a landmass of 238 km^2^ and its headquarter is located at Onireke [11].

### 2.6 Inclusion criteria for selected shopping malls

i. Shopping malls to be used are defined as enclosed buildings with shops representing merchandisers with interconnecting walkways that enable customers to walk from unit to unit with a central management.
ii. The shopping malls must have been functional for at least 6 months.

### 2.7 Exclusion criteria for shopping malls

i. The shopping malls that are not in an enclosed building with shops representing merchandisers with interconnecting walkways that enable customers to walk from unit to unit with a central management were excluded.
ii. The shopping malls that are not functional for at least 6 months were not selected.

### 2.8 Data collection

The on-site observation was performed using observational checklist which was developed and adopted from reviewed journals [12,13]. It was used to assess the conditions of the respective malls. The observational checklist has the following sections:

• **Section A-** (Built environment characteristics and conditions) which assess the facilities and their adequacies
• **Section B-** (Work Conditions) which assess the work conditions in the shopping malls
• **Section C-** (Sanitary conditions of the malls) which assess the sanitary conditions of the shopping malls
• **Section D-** (Waste management Facilities) which assess the waste management facilities in the shopping malls.
• **Section E-** (Source of Air Pollution) which assess the air pollution sources in the selected shopping malls.
• **Section F-** (Safety measures and equipment) which entails the availability of safety measures and equipment in the shopping malls.

Following the observational checklist, the variables of interest were identified as present or absent accordingly.

## 3 Results

### 3.1 Sanitary conditions in shopping malls

Table 1 depicts the criteria that constitute the sanitary conditions in the selected shopping mall. Across the three shopping malls, Mall Q, Mall R and Mall S, toilet facilities were present and functional (++). However, toilet papers and hand washing facilities were available (++) across the selected shopping malls.

**Table 1:** Sanitary Conditions in shopping malls.

Criteria	Mall Q			Mall R			Mall S		
	Absent (−)	Present but non-functional (+)	Present and functional (++)	Absent (−)	Present but non-functional (+)	Present and functional (++)	Absent (−)	Present but non-functional (+)	Present and functional (++)
Toilet facilities in the malls	**NA**	**NA**	**++**	**NA**	**NA**	++	**NA**	**NA**	++
Clean and tidy toilet area	**NA**	**NA**	**++**	**NA**	**NA**	++	**NA**	**NA**	++
Adequate soap for washing hands after use?	**NA**	**NA**	**++**	**NA**	**NA**	++	**NA**	**NA**	++
Availability of toilet papers	**NA**	**NA**	**++**	**NA**	**NA**	++	**NA**	**NA**	++
Availability of hand washing facilities	**NA**	**NA**	**++**	**NA**	**NA**	++	**NA**	**NA**	++
Stores for Cleaning equipment	**NA**	**NA**	**++**	**NA**	**NA**	++	**NA**	**NA**	++
Availability of cleaning equipment	**NA**	**NA**	**++**	**NA**	**NA**	++	**NA**	**NA**	++

Key: Absent (−); Present but non-functional (+); Present and Functional (++); Not Applicable (N).

### 3.2 Waste management facilities in shopping malls

Table 2 shows the waste management facilities available across the shopping malls selected for this study. The basis of categorization of waste management facilities was that Mall with; one to fifteen functional waste bins were categorized as “mildly present”, sixteen to thirty functional waste bins as “moderately”, and over thirty functional waste bins as “highly present”. Waste bins inside the malls were mildly present (+), moderately present (+) and highly present (+++) in Mall Q, Mall R and Mall S respectively. There was absence of overfilled waste bins (−) across the shopping malls but uncovered waste bins were moderately present (++) in Mall Q and Mall R but was absent in Mall S. The emptying of waste bins regularly was highly present (+++) in Mall S but mildly and moderately present in Mall R and Mall Q.

**Table 2:** Waste management facilities across the study locations.

Variable	Mall Q				Mall R				Mall S			
	Absent (−)	Mildly present (+)	Moderately present (++)	Highly present (+++)	Absent (−)	Mildly present (+)	Moderately present (++)	Highly present (+++)	Absent (−)	Mildly present (+)	Moderately present (++)	Highly present (+++)
Waste/dust bins	NA	NA	NA	+++	NA	NA	++	NA	NA	NA	NA	+++
Waste bins inside the mall	NA	+	NA	NA	NA	NA	++	NA	NA	NA	NA	+++
Waste bins outside the mall	NA	NA	NA	+++	NA	+	NA	NA	NA	NA	++	NA
Waste bin uncovered	NA	NA	++	NA	NA	NA	++	NA	–	NA	NA	NA
Waste bins over filled	–	NA	NA	NA	NA	–	NA	NA	–	NA	NA	NA
Littering of refuse around the bins	–	NA	NA	NA	–	NA	NA	NA	–	NA	NA	NA
Waste bins emptied regularly	NA	NA	++	NA	NA	+	NA	NA	NA	NA	NA	+++
Presence of pests in the mall	NA	+	NA	NA	NA	NA	++	NA	NA	+	NA	NA
Presence of stagnant water	–	NA	NA	NA	–	NA	NA	NA	–	NA	NA	NA

Key: Absent (−); Mildly Present (+); Moderately Present (++); Highly Present (+++); Not Applicable (N).

### 3.3 Built environment conditions of the selected shopping malls

Table 3 shows the built environment characteristics of the selected shopping malls. It was observed that there were mold growths on walls and on ceilings in Mall Q and Mall R respectively. However, the three shopping malls had air vents that were free from dusts, properly spaced stairways and handrails, unbroken windows to allow sufficient lightning and presence of emergency exits across the facilities. In Mall R is the only shopping mall out of the selected shopping malls that possesses slippery floors. Although, Mall Q possessed functional escalators and elevators, Mall R and Mall S possessed escalators and elevators respectively.

**Table 3:** Built environment Characteristics of the selected shopping malls.

Characteristics	Mall Q		Mall R		Mall S	
	Absent (−)	Present (+)	Absent (−)	Present (+)	Absent (−)	Present (+)
Intact/undamaged Ceiling Boards	NA	**+**	–	NA	NA	+
Mold Growth on Ceiling	**-**	NA	NA	+	–	NA
Cracks on Walls	**-**	NA	–	NA	–	NA
Dusty/clogged Air Vents	**-**	NA	–	NA	–	NA
Functional Air Conditioning systems	NA	**+**	NA	+	NA	+
Floor Without Cracks	NA	**+**	NA	+	NA	+
Mold Growth On Walls	NA	**+**	–	NA	–	
Unbroken Window Panes In Appropriate Position To Provide Adequate Lightning	NA	**+**	NA	+	NA	+
Sound systems placed in the appropriate positions	NA	**+**	NA	+	NA	+
Proper outdoor floor finishing	NA	**+**	NA	+	NA	+
Properly spaced stairways and hand railings	NA	**+**	NA	+	NA	+
Emergency exits	NA	**+**	NA	+	NA	+
Pedestrians sidewalks	NA	**+**	NA	+	NA	+
Slippery floors	**-**	NA	NA	+	–	NA
Escalators	**NA**	+	NA	+	–	NA
Elevators	**NA**	+	–	NA	NA	+

Key: Absent (−); Present (+); Not Applicable (NA).

### 3.4 Safety measures and equipment in shopping malls

Table 4 shows the various safety measures and equipment across the three shopping malls. Safety signals are present and functional in all the shopping malls except in Mall R in which such safety signals are absent (−). However, first aid boxes, fire extinguishers, fire alarms, security posts, proper scrutiny and checks and surveillance cameras are all present and functional (++) in all the selected shopping malls but smoke detectors are present and functional in all the shopping malls except in Mall R in which this feature is absent (−).

**Table 4:** Safety measures and equipment across study locations.

Variable	Mall Q			Mall R			Mall S		
	Absent (−)	Present but not functional (+)	Present and functional (++)	Absent (−)	Present but not functional (+)	Present and functional (++)	Absent (−)	Present but not functional (+)	Present and functional (++)
Safety signals in place	NA	NA	++	–	NA	NA	NA	NA	++
First aid boxes	–	NA		NA	NA	++	NA	NA	++
Fire extinguishers	NA	NA	++	NA	NA	++	NA	NA	++
Smoke detectors	NA	NA	++	–	NA		NA	NA	++
Fire alarms	NA	NA	++	NA	NA	++	NA	NA	++
Security posts	NA	NA	++	NA	NA	++	NA	NA	++
Proper checks and scrutiny	NA	NA	++	NA	NA	++	NA	NA	++
Surveillance/security cameras	NA	NA	++	NA	NA	++	NA	NA	++

Key: Absent (−); Present but non-functional (+); Present and Functional (++); Not Applicable (NA).

### 3.5 Sources of air pollution

Table 5 shows the sources of air pollution in selected shopping malls. Mall R and Mall S are 5 m within the proximity of major roads and highways and also 5 m within the proximity of the parking lots. However, Mall S is 5 m within the proximity of a public canal, Mall R within 5 m within the proximity of a public drainage and gutters while Mall Q possess flora within the mall while flora were present outside the three selected malls.

**Table 5:** Sources of air pollution across the study locations.

Criteria	Mall Q		Mall R		Mall S	
	Absent (−)	Present (+)	Absent (−)	Present (+)	Absent (−)	Present (+)
Proximity of the major road and highways within 5 m to the malls	–	NA	NA	+	NA	+
Proximity of parking lot to the mall within 5 m to the mall	–	NA	NA	+	NA	+
Proximity of the public drainage/gutters within 5 m to the malls	–	NA	NA	+	–	NA
Proximity of canals within 5 m to the mall	–	NA	–	NA	NA	+
Proximity of mechanic workshops within 5 m to the mall	–	NA	NA	+	NA	+
Presence of flora outside the mall	NA	+	NA	+	NA	+
Presence of flora inside the mall	NA	+	–	NA	–	NA
Presence of fauna (animals) outside the mall	NA	+	–	NA	–	NA
Air fresheners/insect repellants	NA	+	NA	+	NA	+

## 4 Discussion

From the sanitary inspection across the malls in this study, there were mold growths on walls and ceilings in Mall Q and Mall R respectively. Moss growth is subject to outdoor environments except in case of a water leak [14]. The mold growth on the ceiling in Mall R was due to the damaged ceiling boards and water leakages. According to previous studies, many fungi grow indoors as mold where moisture is present. Some of these fungi can cause toxic reactions and may lead to infections in susceptible individuals and excessive mold growth can contribute to respiratory illness and infections [15,16].

As regards waste management, waste bins inside the malls were mildly present, moderately present and highly present in Mall Q, Mall R and Mall S, respectively. There is growing pressure for shop centers and shopping malls to manage their waste sustainably by acting responsibly and thoroughly. Moreover, the practical significance of adhering to waste regulations and environmental standards has led the majority of shopping malls to value facilities management services [21]. Another research at a UK shopping Centre reveals little effort has been taken to research the current recycling of the solid waste in the UK shopping center industry. The majority of prior research on waste minimization through recycling has focused on municipal buildings, commercial office buildings, medical facilities, hotels, and educational and industrial buildings [22].

In both European nations and the United States, there is an excessive reliance on landfill disposal. Nevertheless, in many developing countries like Nigeria where this study was conducted, waste disposal conditions remain primitive. Although garbage disposal generates the potential for various severe health and environmental effects, including emissions to air, surface water, and groundwater, it entirely depends on how it is managed [23]. Subsequently, there was an absence of overfilled waste bins across the shopping malls in this study. Still, uncovered waste bins were moderately present in Mall Q and Mall R but absent in Mall S. The emptying of waste bins regularly was highly present in Mall S, which shows proper waste disposal in the mall.

Subsequently, several previous studies associated respiratory health effects with dampness and mold in homes and moist walls in public structures [17]. This agrees with a previous study on shopping malls in Singapore which shows how molds can threaten human life. The study reveals a collection of 40 samples from the 15 shopping centers and analyzed by a DNA-based technology called mold-specific quantitative PCR (MSQPCR). Mold was detected at some concentrations and many were much more abundant than the average in the shopping centers [18]. Furthermore, a Nordic multidisciplinary committee reported the findings of a review on dampness in buildings (including exposure to mites) and health, with the conclusions that dampness in buildings appears to increase the risk for health effects in the airways, such as cough, wheeze and asthma evidence for a causal association between ‘dampness’ and health effects is strong [19]. It will be appropriate to say that the leakage in Mall R is responsible for the slippery floor, which is the only Mall with a slippery floor in this survey.

The three shopping malls in this study had air vents free from dust. Also, they had adequately spaced stairways, handrails, and unbroken windows to allow sufficient lightning and the presence of emergency exits across the facilities. Nevertheless, this does not keep outdoor air pollutants from getting inside the Mall, as most malls are close to the road. A study revealed in the city of Changsha, China, revealed the impact of outdoor pollutants on indoor air quality in shopping malls, with an indoor-to-outdoor ratio (I/O ratio) of Particulate Matter (PM_2.5_) concentrations in a shopping mall ranging from 0.46 to 0.52 [20]. Subsequently, the glass door of these malls’ entrances is open from time to time based on the entry and exit of visitors, which may introduce the outdoor air pollutants to the indoor environment. The limitation in this study is that the outdoor and indoor air quality ratio of the malls was not measured.

Visitors’ perceptions of a shopping mall’s safety are influenced by several overlapping factors, including the facility’s safety, the quality and maintenance of the shopping mall environment, and its surroundings [24]. Safety signals are present and functional in all the shopping malls except in Mall R, in which such safety signals are absent. However, first aid boxes, fire extinguishers, fire alarms, security posts, proper scrutiny and checks and surveillance cameras are all present and functional in all the selected shopping malls. Smoke detectors are present and functional in all the shopping malls except in Mall R, in which this feature is absent. Interestingly, previous research shows these factors (surveillance camera, emergency exits and fire-extinguisher) impact safety in a shopping mall and shoppers’ health [25].

Mall R and Mall S are 5 m within the proximity of major roads and highways and 5 m within the proximity of the parking lots. However, Mall S is 5 m within the proximity of a public canal, and Mall R is within 5 m within the proximity of public drainage and gutters. At the same time, Mall Q possesses flora within the Mall while flora was present outside the three selected malls. Shopping malls in Hong Kong are usually located near major roads. Therefore, indoor air quality (IAQ) in these buildings is subject to the infiltration of outdoor traffic-related pollutants. Exposure to particles and gaseous air pollutants is associated with many adverse health effects. Similarly, it has been discovered that air pollution in China during wintertime consistently draws media attention [26]. Therefore, countermeasures by individuals may be natural.

## 5 Conclusion

This research has shown that certain shopping malls in Nigeria possess more health and environmental hazard on people than others, especially the ones constructed along road sides. Since malls are normally located close to main roads in urban cities, vehicle exhausts, dusts, concentrations of Carbon dioxides, and several other pollutants affect the quality of air in malls. Even though the three shopping malls in this research had air vents free from dust, this does not keep outdoor air pollutants from getting inside the Mall. The glass doors of the entrances to these malls are opened from time to time based on the entry and exit of visitors, which introduces the outdoor air pollutants to the indoor environment of the malls. Further research could be done to measure the quality of outdoor and indoor ratio of these malls. More so, mold growth was present in Mall R due to damaged ceilings and water leakages. This fungus particularly causes toxic reactions and may lead to respiratory illness and infections. More so, there should be improvement on the hygiene and sanitary conditions within the malls and proper housekeeping practices should be encouraged to prevent the proliferation of algal growth on walls and roofs of shopping malls. Lastly, trainings, seminars and workshops on safety practices and effects of poor indoor air quality on health should be encouraged. This will help check out possible sources of health hazards for mall operators and the general public.

Consequently, we recommend that there should be periodic environmental monitoring of shopping malls in Nigeria by certified professionals. This will protect the health of the public and most importantly, the health of operators in malls. Constant monitoring and evaluation will reduce the risk of constructing malls within areas of predominant air pollutants and reduce the risk of leaving damaged mall facilities unattended to.

## Ethical approval

Ethical approval was obtained from the University of Ibadan/University College Hospital Institutional Ethics Committee and Review Board with the reference number “UI/EC/19/0142”.

## Funding

This study did not receive any specific grant from funding agencies in the public, commercial, or not-for-profit sectors. The authors wish to acknowledge the Management of Afe Babalola University for their support for the payment of the APC.

## Declaration of competing interest

The authors hereby declare there is no conflict of interest associated with this study or any of the procedures and materials used for the purpose of the study.
